# Development and validation of a 21-gene prognostic signature in neuroblastoma

**DOI:** 10.1038/s41598-023-37714-9

**Published:** 2023-08-02

**Authors:** Mehul Gupta, Sunand Kannappan, Mohit Jain, David Douglass, Ravi Shah, Pinaki Bose, Aru Narendran

**Affiliations:** 1 Department of Pediatrics and Oncology, Cumming School of Medicine, University of Calgary, 3330 Hospital Drive NW, Calgary, AB T2N 4N1 Canada; 2 Department of Pediatrics, Hematology/Oncology Section, Arkansas Children’s Hospital, University of Arkansas for Medical Sciences, Little Rock, AR 72202 USA; 3 Departments of Oncology and Biochemistry and Molecular Biology, Cumming School of Medicine, University of Calgary, 3330 Hospital Drive NW, Calgary, AB T2N 4N1 Canada; 4 Cumming School of Medicine, Arnie Charbonneau Cancer Institute, University of Calgary, Calgary, AB T2N 4N1 Canada

**Keywords:** Cancer, Computational biology and bioinformatics, Genetics, Molecular biology

## Abstract

Survival outcomes for patients with neuroblastoma vary markedly and reliable prognostic markers and risk stratification tools are lacking. We sought to identify and validate a transcriptomic signature capable of predicting risk of mortality in patients with neuroblastoma. The TARGET NBL dataset (n = 243) was used to develop the model and two independent cohorts, E-MTAB-179 (n = 478) and GSE85047 (n = 240) were used as validation sets. EFS was the primary outcome and OS was the secondary outcome of interest for all analysis. We identified a 21-gene signature capable of stratifying neuroblastoma patients into high and low risk groups in the E-MTAB-179 (HR 5.87 [3.83–9.01], *p* < 0.0001, 5 year AUC 0.827) and GSE85047 (HR 3.74 [2.36–5.92], *p* < 0.0001, 5 year AUC 0.815) validation cohorts. Moreover, the signature remained independent of known clinicopathological variables, and remained prognostic within clinically important subgroups. Further, the signature was effectively incorporated into a risk model with clinicopathological variables to improve prognostic performance across validation cohorts (Pooled Validation HR 6.93 [4.89–9.83], *p* < 0.0001, 5 year AUC 0.839). Similar prognostic utility was also demonstrated with OS. The identified signature is a robust independent predictor of EFS and OS outcomes in neuroblastoma patients and can be combined with clinically utilized clinicopathological variables to improve prognostic performance.

## Introduction

Neuroblastoma is the most common extracranial solid tumour in children accounting for approximately 7–10% of all childhood cancers^1–3^. Neuroblastoma is characterized by substantial heterogeneity in tumour characteristics^4–6^ and patient outcomes, ranging from spontaneous regression in some patients^7^ to metastatic treatment-resistant disease in others^8^. Given this heterogeneity, multiple staging systems have been developed to stratify risk for patients diagnosed with neuroblastoma. The International Neuroblastoma Staging System (INSS) is a postsurgical staging system, developed in 1986, that utilizes the disease location, lymph node status, and the extent of surgical resection for patient classification^9,10^. The INSS has been largely supplanted by the International Neuroblastoma Risk Group Staging System (INRGSS), which was formed in 2005 to create a staging classification independent of the findings from surgical resection. This tool utilizes the presence of image defined risk factors (IDRF) to categorize locoregional tumors as L1 (IDRF absent) or L2 (IDRF present)^11,12^. Both of these tools have consistently demonstrated a strong association with survival outcomes^11–13^.

In addition to these well-established staging systems numerous studies have identified other clinical, pathologic, and genomic characteristics associated with survival in neuroblastoma. For example, patient age at diagnosis represents an important prognostic variable, with older patients (often defined as those greater than 18 months of age) consistently being shown to experience worse outcomes^8,14^. MYCN copy number amplification has been shown to be independently associated with poor clinical outcomes and is found in approximately 25% of neuroblastoma cases and 40% of high-risk cases^15^. Other segmental chromosomal aberrations such as chromosome 1p deletion, 11q deletion or 17q gain have also been associated with poor survival outcomes in neuroblastoma^16,17^. Additionally molecular and pathologic features such as histologic category^18^, DNA ploidy^19^ and grade of tumor differentiation have been identified as prognostic markers in neuroblastoma.

Therefore, current management approaches largely rely on risk classification scores that incorporate the INRGSS staging system along with a combination of the clinical, pathologic, and genomic characteristics that have shown an association with survival in neuroblastoma. Among the most well recognized of these scores is the 2021 revised Children’s Oncology Group (COG) risk classification criteria^8^. This scoring system utilizes INRGSS stage, as well as age at diagnosis, histologic classification and the presence of molecular and pathologic biomarkers such as MYCN amplifications status, DNA ploidy, and segmental chromosomal aberrations to categorize patients into low, intermediate and high-risk group^8,20^. A majority of patients classified as low-risk can be observed for spontaneous tumor regression or treated with surgical resection alone^21^. Those with intermediate-risk disease are often treated with neoadjuvant multi-agent chemotherapy followed by surgical resection^21^. High-risk patients receive an intensive treatment regimen including an induction phase with multi-agent chemotherapy and surgical resection, a consolidation phase of high-dose chemotherapy with autologous stem cell rescue and radiotherapy, and a post-consolidation phase where patients often receive immunotherapy to target minimal residual disease, in an effort to prevent relapse^22^. Outcomes vary considerably between groups, with low risk patients experiencing a 5 year overall survival of 98% compared to 62% of those with high-risk disease^8^. Due to this substantial prognostic difference, a great deal of effort has been directed towards deintensification of treatment for low and intermediate-risk patients^23,24^, and intensification of therapy for high-risk patients^25–27^to improve outcomes in neuroblastoma.

In addition to the improving the efficacy of therapeutic approaches, efforts to improve patient outcomes in neuroblastoma have focused on development of clinical and molecular risk stratification tools to inform clinical decision-making^28^. In particular, improved stratification of neuroblastoma patients may allow for intensification and deintensification of treatment for the appropriate patients, minimizing toxicity while maximizing therapeutic benefit^21^. This is particularly important in neuroblastoma, given that pediatric populations are at risk of late complications of toxicity from chemotherapy and radiation therapy regimens during early development^29^.

Substantial work has gone into the development of novel molecular biomarkers to predict prognosis of neuroblastoma patients, including studies of long noncoding RNAs^30,31^, mircoRNAs^32,33^, and genomic aberrations^34^. However, the most well studied category of prognostic biomarkers for neuroblastoma utilize high throughput transcriptomic sequencing technologies to identify gene expression-based predictors known as gene signatures. Many studies have attempted to generate such signatures in neuroblastoma^35–38^, varying substantially with regards to the statistical techniques used, rigor of external validation, quantification platforms used, and number of transcripts included. Many of the previous studies attempting to develop prognostic signatures for neuroblastoma have 2 incorporated biological insights into the selection of prognostic genes. Some studies utilize differential expression analysis, or ontological groups of genes implicated in signaling pathways, tumorigenesis, or neuroblastoma aggressiveness to restrict their pool of candidate transcripts^39–42^. While these approaches are biologically informed and may drive improved understanding of the biology underpinning neuroblastoma, limiting the selection of genes in this way may exclude those with prognostic importance that are not currently associated with biological processes implicated in neuroblastoma. Additionally, only a small number of these signatures have been developed and validated across different transcript quantification platforms, limiting the generalizability of the tools developed^43^. Moreover, clinical translation of many of these proposed signatures may be difficult given the large number of transcripts included which would require costly and complex analysis, or the lack of a proposed mechanism to help clinicians incorporate existing risk stratification techniques with the generated signatures to create more nuanced risk groups^44^. As a result of these factors, there are no transcriptomic signatures for neuroblastoma patients currently used in the clinical setting.

In this study, using a biologically unbiased machine learning approach, we developed and externally validated a 21-gene transcriptomic signature predictive of overall survival (OS) and event-free survival (EFS) for neuroblastoma patients. In multivariate analysis with relevant clinical covariates, our signature remained an independent prognostic factor. Finally, we built a clinically translatable risk-stratification model by combining our 21-gene signature with clinical and molecular features currently used for prognostication in neuroblastoma.

## Methods

### Patient cohorts

A two-phase study design was utilized, with the initial discovery phase consisting of developing a prognostic signature via *in-silico* analysis of microarray expression data, and a subsequent validation phase being used to ascertain the prognostic utility of the signature in two external, independent cohorts.

The discovery dataset consisted of patients enrolled in the Therapeutically Applicable Research to Generate Effective Treatments (TARGET) initiative (n = 249) neuroblastoma study (sub-study ID phs000467). The samples analyzed in this cohort consist of optimal cutting temperature (OCT) embedded primary tumor samples collected at the time of diagnosis from patients enrolled in COG studies and clinical trials. Samples were prepared and transcription was quantified as described by the TARGET consortium^45^. In brief, transcript quantification was performed on an Affymetrix Human Exon ST 1.0 microarray and scanned as per manufacturer’s instructions. Transcript data was normalized and summarized using rma-sketch analysis (which approximates quantile normalization) using Affymetrix power tools. Probe sets with low expression and low variation were removed and the resulting probe sets were averaged by transcript identification based on Affymetrix annotations. Clinical and transcriptomic data was obtained from the TARGET neuroblastoma repository available at https://portal.gdc.cancer.gov/projects.

Two independent cohorts were utilized for validation of the signature generated in the discovery dataset, E-MTAB-179 and GSE85047. The E-MTAB-179 cohort (n = 478) consists of snap-frozen primary neuroblastoma tissue samples obtained prior to cytotoxic treatment as described previously^46^. This cohort quantified transcript expression utilizing a custom Agilent Neuroblastoma array (A-MEXP-1746). Clinical and transcriptomic data for this cohort was obtained from the European Bioinformatics Institute ArrayExpress platform (accession ID E-MTAB-179). Transcriptomic data was normalized using a quantile algorithm. The GSE85047 cohort (n = 283) consists of primary neuroblastoma tissue samples obtained prior to treatment from the Neuroblastoma Research Consortium (NRC) and was processed as described previously^47^. Transcript quantification in this cohort was performed using a Affymetrix Human Exon ST 1.0 microarray. Downloaded transcript data was normalized and summarized using rma-sketch analysis, which approximates quantile normalization, using Affymetrix power tools. Expression and clinical data for this cohort was obtained from the Gene Expression Omnibus (accession ID GSE85047).

Candidate transcripts in the discovery cohort were filtered to include only those present across all discovery and validation cohorts, to ensure any generated prognostic signature could be validated (n = 1780). Stage 4 s patients were excluded from further analysis (TARGET n = 0, GSE85047 n = 27, E-MTAB-179 n = 62) as the unique genetic, epigenetic, and transcriptomic characteristics that drive spontaneous regression and improved outcomes in these patients may have interfered with the selection of candidate transcripts that were broadly prognostic across other stages of neuroblastoma^48^. Additionally, those with missing survival and/or pathological variables (TARGET n = 6, GSE85047 n = 16, E-MTAB-179 n = 0) were also excluded from further analysis. Following exclusions, a total of 243 patients were included in the TARGET cohort, 240 in the GSE85047 cohort, and 416 in the E-MTAB-179 cohort for further analysis. Clinicopathological and patient demographic data for the cohorts is shown (Table 1).

**Table 1:** Clinical and pathological characteristics of neuroblastoma patients included in study cohorts: TARGET NBL, GSE85047, and E-MTAB-179.

	TARGET NBL	GSE85047	E-MTAB-179
	(n = 243)	(n = 240)	(n = 416)
MYCN amplification status			
Amplified	68 (28.0%)	53 (22.1%)	64 (15.4%)
Not Amplified	175 (72.0%)	187 (77.9%)	352 (84.6%)
INSS Stage			
Stage 1	30 (12.3%)	43 (17.9%)	119 (28.6%)
Stage 2	0 (0%)	34 (14.2%)	80 (19.2%)
Stage 3	1 (0.4%)	42 (17.5%)	69 (16.6%)
Stage 4	212 (87.2%)	121 (50.4%)	148 (35.6%)
Age at diagnosis (days), median (range)	1040 (6–7450)	565 (0–7100)	459 (0–8980)
Follow-up for overall survival (days), median (range)	1560 (2–5560)	1230 (1–6460)	1380 (8–6600)
Overall survival events	137 (55.6%)	72 (30.0%)	87 (20.9%)
Follow-up for Event-free survival (days), median (range)	715 (2–5560)	813 (1–6460)	1080 (8–6600)
Event-free survival events	134 (55.1%)	94 (39.2%)	140 (33.7%)

Data are n (%) unless otherwise indicated.

### Prognostic model generation

To create a prognostic model from the TARGET neuroblastoma cohort expression profiles, Least Absolute Shrinkage and Selection Operator (LASSO) regularized regression employing Cox Proportional Hazards (CoxPH) was utilized (available through the glmnet R package). This technique is a biologically unbiased machine learning model that is useful in cases where the number of predictors is much larger than the number of observations, and thus is particularly well suited to transcriptomic data. 20-fold cross validation with partial-likelihood deviance loss was performed to obtain the optimal lambda (0.1006), which represents the regularization parameter utilized in the creation of the final model. Following lambda tuning, the model was generated with an alpha of 1 to limit the number of features selected. EFS was used as the primary endpoint to generate the prognostic model.

### Signature validation and survival analysis

EFS was used as the primary endpoint, and OS as the secondary endpoint for all statistical analyses. The model weights obtained in the discovery phase of the study were used in all subsequent validation analyses to prevent overestimation of effect caused by retraining of the signature. Univariate CoxPH regression analysis was used to estimate the hazard ratio (HR) to evaluate the association of the obtained prognostic signature with EFS and OS in discovery and validation cohorts. Median signature risk score was used to subgroup patients into low and high-risk groups in all analyses. EFS and OS curves were visualized using Kaplan–Meier curves. 5 year receiver operator characteristic (ROC) curves were constructed to evaluate the sensitivity and specificity of the prognostic signature in predicting survival outcomes. 95% confidence intervals are reported for regression analysis as well as on ROC and Kaplan–Meier curves.

To evaluate the performance of the prognostic signature within clinically important subgroups, patients were stratified based on age at diagnosis (> 18 months or < 18 months), MYCN amplification status (amplified or unamplified), and INSS stage (I/II or III/IV). Once stratified, patients in each subgroup were divided into high and low-risk groups by the median prognostic signature risk score, and univariate CoxPH regression was performed to assess the prognostic utility of the signature in these subgroups.

To assess the independent prognostic value of the signature, available clinicopathological variables were dichotomized: age at diagnosis (> 18 months vs. < 18 months), MYCN amplification status (amplified vs. unamplified), and INSS stage (I/II vs. III/IV). Univariate CoxPH regression was performed on clinicopathological variables to assess their association with survival outcomes, with variables significantly associated being retained for multivariate CoxPH regression with the dichotomized scores for the 21-gene signature.

### Combined risk-stratification model generation

Both validation cohorts were pooled together, and dichotomized clinicopathological variables and signature risk were regressed in multivariate CoxPH analysis. This multivariate model was then used to generate nomograms for the prediction of 5-year survival outcomes. These nomograms generated a combined risk score incorporating the signature risk group as well as prognostic clinicopathologic variable status. Kaplan–Meier curves were plotted to visualize survival differences between high and low-risk groups using the median combined risk score as cut-off; univariate CoxPH analysis was used to assess the association of the combined risk score with survival. ROC curves were also generated to ascertain the sensitivity and specificity for prediction of 5-year survival outcomes for the combined risk score in comparison to dichotomized clinicopathologic variables or the prognostic signature alone.

All statistical analyses were performed using R (version 3. 4. 1; http://www.r-project.org/). All reported P values are two-tailed, and an alpha of 0.05 was used as the threshold for statistical significance.

## Results

### A 21-gene signature for survival risk stratification of neuroblastoma patients

**Figure 1:**
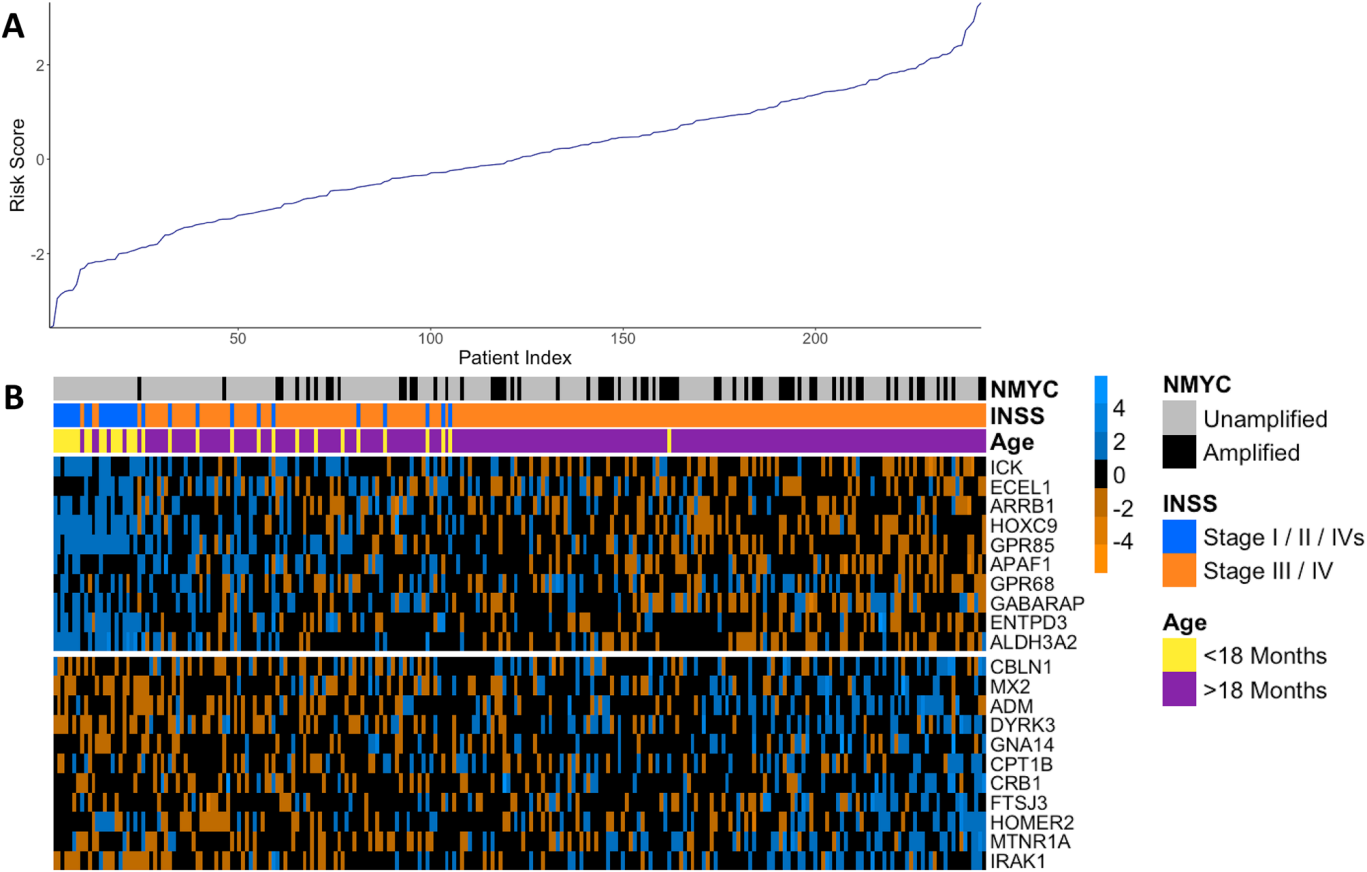
Expression heat map of genes included in the 21-gene prognostic signature. Each column represents one patient in the TARGET neuroblastoma discovery cohort (n = 243), and each row represents a gene included in the model. Patients are arranged from low risk (left) to high risk (right) based on the 21-gene risk score and genes are arranged from highest positive association with the risk (bottom) to highest negative association with the risk (top). The line plot demonstrates the risk score of patients as calculated by the 21-gene prognostic signature, with low values associated with low risk and high values associated with high risk (**A**). For the heatmap, blue represents low expression and orange represents high expression. Genes above the separation are positively associated with risk, and genes below the separation are negatively associated with risk. Clinical variables including MYCN amplification status, INSS stage and age at diagnosis are shown as annotations for each patient in the sample (**B**).

**Figure 2:**
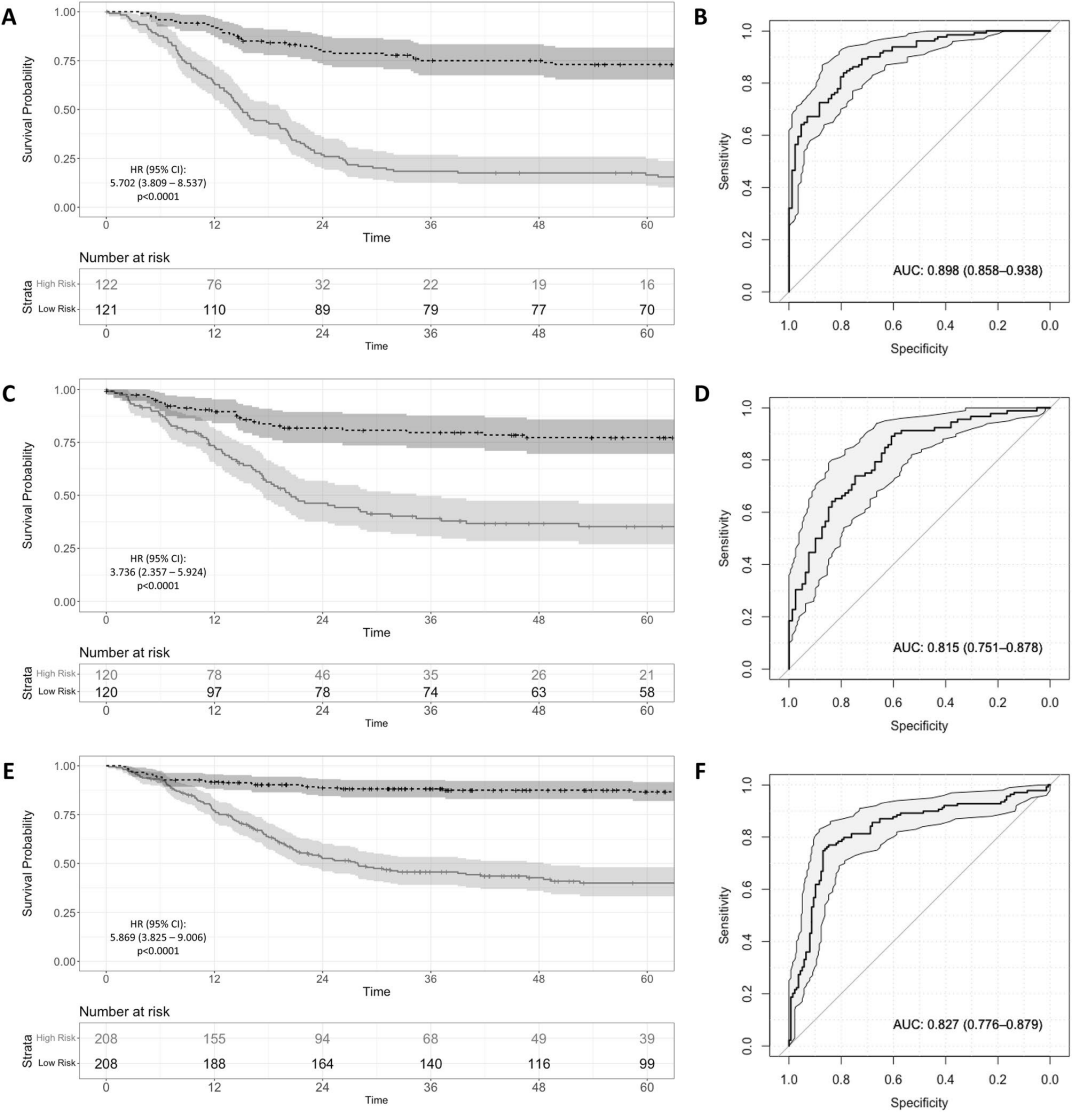
Event-free survival stratified by 21-gene prognostic signature risk score in discovery and validation cohorts. Kaplan Meier survival curves with cox proportional hazards analysis showing risk of event-free survival in low risk (black curve) and high risk (grey curve) groups generated using a median cut-off for the 21-gene risk score and ROC curve analysis demonstrating the predictive capacity of the risk score for 5 year event-free survival in the discovery cohort (Panel **A** and **B**), GSE85047 validation cohort (Panel **C** and **D**), and E-MTAB-179 validation cohort (Panel **E** and **F**).

LASSO penalized regression utilizing the CoxPH model in the TARGET discovery cohort identified a set of 21 genes significantly associated with EFS in neuroblastoma patients (Fig. 1). Of the genes included in the signature, 10 were associated with improved prognosis, and 11 were associated with poor prognosis (Supplementary Table 1). The signature effectively discriminated between patients with good versus worse prognosis in the discovery cohort (TARGET HR 4.20 [2.89–6.10], *p* < 0.0001) (Fig. 2A). These findings were validated in multiple validation cohorts, where significant differences in EFS were observed based upon the signature risk score (GSE85047: HR 4.20 [2.82–8.65], *p* < 0.0001; E-MTAB-179: HR 5.87 [3.83–9.01], *p* < 0.0001) (Fig. 2B,C). 5 year ROC curves in discovery and validation cohorts also demonstrated a substantial ability to predict EFS for neuroblastoma patients (TARGET AUC = 0.898; GSE85047 AUC = 0.815; E-MTAB-179 AUC = 0.827) (Fig. 2D,F). Similar associations between the generated signature and OS were also observed. The 21-gene signature was able to stratify OS across discovery (TARGET HR 4.20 [2.89–6.10], *p* < 0.0001) and validation cohorts (GSE85047; HR 4.941 [2.823–8.647], *p* < 0.0001; E-MTAB-179; 37.240 [11.760–117.900], *p* < 0.0001) (Supplementary Fig. 1 A-C). Similarly, 5 year ROC curves demonstrated substantial predictive utility of the 21-gene signature for OS (TARGET AUC = 0.840; GSE85047 AUC = 0.833; E-MTAB-179 AUC = 0.904) (Supplementary Fig. 1 D-F). These results demonstrate that we have identified a 21-gene signature that robustly predicts both OS and EFS outcomes in neuroblastoma patients.

### The 21-gene signature is predictive of survival within clinically important neuroblastoma subgroups

In addition to predicting survival within unstratified neuroblastoma cohorts, analysis within clinically relevant subgroups was conducted to assess if the identified 21-gene signature could be utilized to stratify these patients more accurately. Patients in validation cohorts were iteratively subdivided based on age at diagnosis (> 18 months or < 18 months), MYCN amplification status (amplified or unamplified), and INSS stage (I/II or III/IV) and univariate CoxPH was used to assess signature association with survival. In both validation cohorts, the 21-gene signature demonstrated significant ability to stratify EFS within age < 18 months at diagnosis, age > 18 months at diagnosis, INSS stage III/IV, and unamplified MYCN status subgroups (Table 2). Similar trends were noted in OS analysis, with the signature able to stratify within age < 18 and > 18 months at diagnosis, INSS stage III/IV and unamplified MYCN status patients in the GSE85047 cohort. In the E-MTAB-179 cohort, due to the absence of OS events in the low-risk strata of the INSS stage I/II and age < 18 months at diagnosis subgroups, these specific analyses could not be performed. However, the signature remained significant within age > 18 months at diagnosis, INSS stage III/IV and unamplified MYCN status groups (Supplementary Table 2). Further analysis in a pooled validation cohort demonstrates that the obtained signature is a strong predictor of EFS in patients with multiple clinicopathologic prognostic factors associated with low-risk (Supplementary Table 3), though similar findings were not replicated in OS given the paucity of events in for this outcome measure (Supplementary Table 4) These findings suggest that the generated 21-gene signature may be useful for identification of neuroblastoma patients at higher and lower risk even within existing clinical subgroups currently used for prognostication.

**Table 2:** Univariate cox proportional hazards analysis of event free survival for high versus. low-risk groups as defined by the 21-gene prognostic signature in clinically relevant subgroups.

		HR (95% CI)	*p*-value
GSE85047 *(N* = *240, events* = *94)*	Age diagnosis	1.608	**0.0500**
	(> 18 Months)	(0.9998–2.587)	
	*n* = *126, events* = *71*		
	Age at diagnosis	5.715	**0.0016**
	(< 18 Months)	(1.942–16.820)	
	*n* = *114, events* = *23*		
	INSS stage	2.026	**0.0017**
	(III, IV)	(1.304–3.149)	
	*n* = *163, events* = *85*		
	INSS stage	3.757	0.0989
	(I, II)	(0.780–18.100)	
	*n* = *77, events* = *9*		
	MYCN	0.932	0.8290
	Amplified	(0.492–1.764)	
	*n* = *53, events* = *38*		
	MYCN	3.652	** < 0.0001**
	Unamplified	(2.015–6.618)	
	*n* = *187, events* = *56*		
E-MTAB-179* (N* = *416, events* = *140)*	Age at Diagnosis	2.050	**0.0007**
	(> 18 months)	(1.352–3.109)	
	*n* = *168, events* = *94*		
	Age at diagnosis	3.203	**0.0005**
	(< 18 months)	(1.657–6.191)	
	*n* = *248, events* = *46*		
	INSS stage	2.801	** < 0.0001**
	(III, IV)	(1.893–4.143)	
	*n* = *217, events* = *115*		
	INSS stage	1.888	0.1280
	(I, II)	(0.834–4.275)	
	*n* = *199, events* = *25*		
	NMYC	1.089	0.7760
	Amplified	(0.606–1.955)	
	*n* = *64, events* = *45*		
	NMYC	5.720	** < 0.0001**
	Unamplified	(3.381–9.677)	
	*n* = *352, events* = *95*		

Subgroups were stratified by age at diagnosis (< 18 months and > 18 months groups), INSS stage (I, II and III, IV groups) and MYCN amplification status (amplified and unamplified groups). Dichotomization of patients into high and low-risk groups was performed using the median prognostic signature score. Significant *P* values are shown in bold.

### 21-gene signature is an independent predictor of survival outcomes in neuroblastoma patients

Univariate and multivariate CoxPH analyses were conducted to compare the signature with various clinicopathological features including age at diagnosis, MYCN amplification status and INSS stage. Patients in validation cohorts were classified into clinicopathological subgroups as before, and high and low risk groups based on the median 21-gene signature risk score. In univariate CoxPH analysis, age > 18 months at diagnosis, INSS stage III/IV, MYCN amplification and high 21-gene risk score were all significant predictors of poor survival in both validation cohorts (*p* < 0.0001 for all analyses) (Table 3). Multivariate CoxPH analysis utilizing features statistically significant in univariate modelling, demonstrated that the identified 21-gene risk score remained an independent prognostic factor for predicting outcomes in neuroblastoma patients. In addition to the prognostic signature, INSS stage and MYCN status also emerged as independent risk factors, while age at diagnosis was only significantly associated in the GSE85047 validation cohort (Table 3). These results indicate that the 21-gene prognostic signature maintained its independent association with EFS when analyzed in multivariate analysis adjusting for significant clinicopathologic covariates. OS analysis revealed similar trends in univariate CoxPH analysis, with all variables being significant (Supplementary Table 5). In multivariate analysis, all variables retained significance in the E-MTAB-179 cohort, however the 21-gene signature was not significantly associated with survival in the GSE85047 cohort (Supplementary Table 5).

**Table 3:** Univariate and multivariate cox proportional hazards analysis of clinicopathologic variables and 21-gene risk score for event-free survival.

		Univariate	*p*-value	Multivariate	*p*-value
		HR		HR	
		(95% CI)		(95% CI)	
GSE85047 *(N* = *240, events* = *94)*	Age at Diagnosis	3.786	** < 0.0001**	1.769	**0.03810**
	*(*> *18 Months vs.* < *18 Months)*	(2.355–6.087)		(1.032–3.033)	
	INSS Stage	6.090	** < 0.0001**	2.950	**0.00595**
	*(III, IV vs. I, II)*	(3.056–12.140)		(1.365–6.378)	
	MYCN Status	3.373	** < 0.0001**	1.676	**0.02464**
	*(Amplified vs. Unamplified)*	(2.226–5.112)		(1.068–2.631)	
	21-gene risk score	3.736	** < 0.0001**	1.772	**0.03608**
	*(High vs. Low)*	(2.357–5.924)		(1.038–3.026)	
E-MTAB-179 *(N* = *416, events* = *140)*	Age at Diagnosis	3.565	** < 0.0001**	1.272	**0.25546**
	*(*> *18 Months vs.* < *18 Months)*	(2.501–5.083)		(0.8402–1.926)	
	INSS Stage	5.172	** < 0.0001**	2.782	** < 0.0001**
	*(III, IV vs. I, II)*	(3.354–7.976)		(1.7385–4.451)	
	MYCN Status	4.025	** < 0.0001**	1.685	**0.00766**
	(Amplified vs. Unamplified)	(2.807–5.771)		(1.1482–2.472)	
	21-gene risk score	5.869	** < 0.0001**	3.024	** < 0.0001**
	(High vs. Low)	(3.825–9.006)		(1.8148–5.040)	

Patients were dichotomized by age at diagnosis (< 18 months and > 18 months), INSS stage (I, II and III, IV) and MYCN amplification status (amplified and unamplified groups). Dichotomization of patients into high and low-risk groups was performed using the median prognostic signature score. Significant *P* values in multivariate analysis are shown in bold.

### Establishment of a nomogram for prediction of survival in neuroblastoma patients

**Figure 3:**
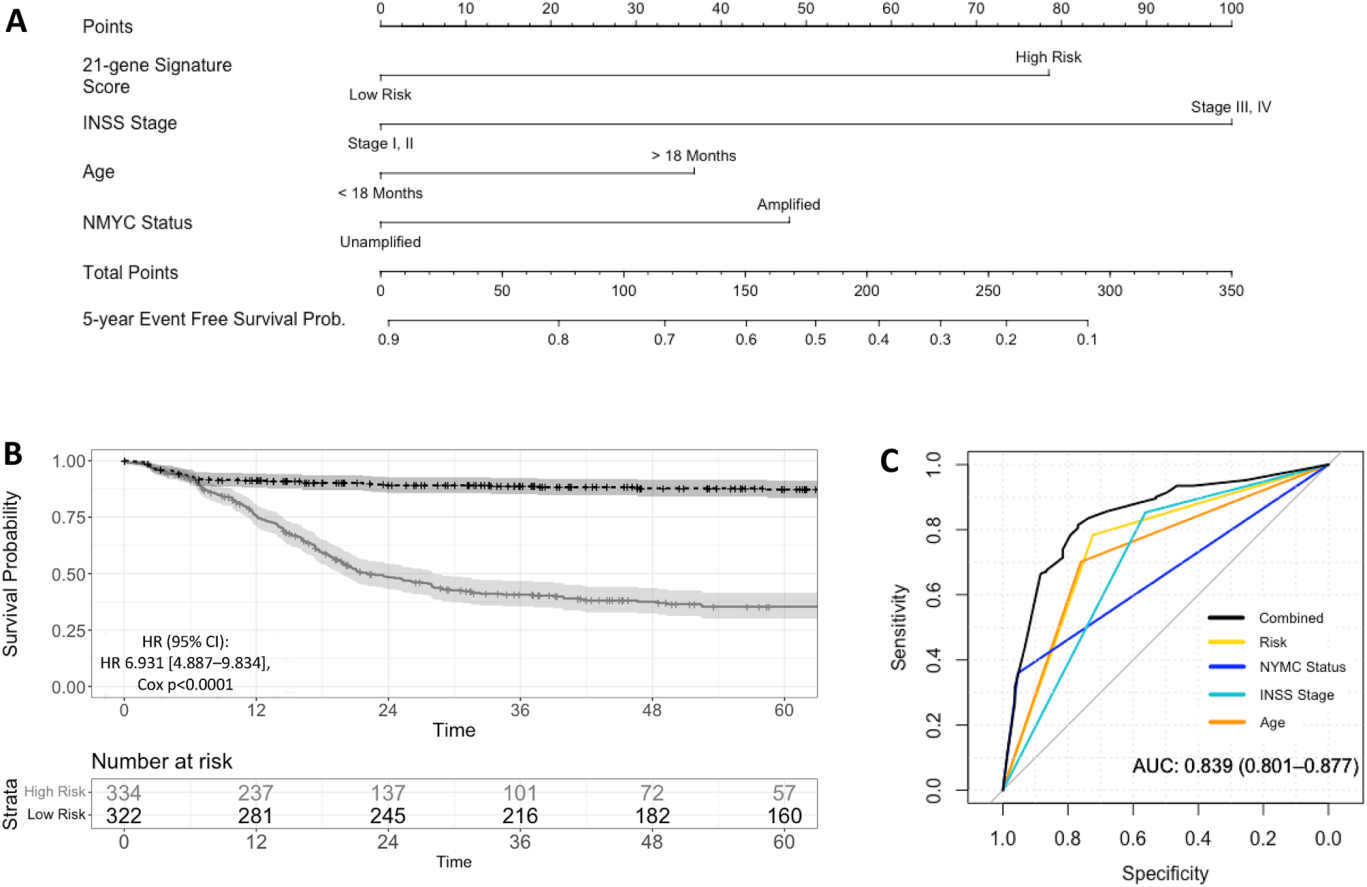
Nomogram integrating 21-gene risk score and other clinicopathological variables for prediction of 5 year event-free survival in a pooled validation cohort. Nomogram incorporating clinicopathological characteristics and 21-gene risk score to generate a combined risk score (Panel **A**). Kaplan Meier survival curves with cox proportional hazards analysis showing risk of event-free survival in low risk (black curve) and high risk (grey curve) groups generated using a median cut-off for the combined prognostic risk score in the pooled validation cohort (Panel **B**). ROC curve analysis demonstrating the predictive capacity of the combined risk score compared to the 21-gene risk score and other clinicopathological variables in isolation for 5 year event-free survival in the pooled validation cohort (Panel **C**).

To improve the performance and clinical utility of the generated 21-gene signature, multivariate regression was used to incorporate the signature with significant clinicopathological variables to generate a combined risk score. Validation cohorts were pooled together for these analyses, and multivariate CoxPH incorporating the 21-gene signature, age at diagnosis, MYCN amplification status, and INSS stage was performed. Analysis in the pooled cohort for EFS and OS demonstrated that validation cohort identity was not associated with survival (EFS *p* = 0.5210, OS *p* = 0.5270), while the 21-gene signature score, age at diagnosis, MYCN status, and INSS stage remained significant. These clinicopathological characteristics and the 21-gene score were used to generate a nomogram giving a combined risk score for 5 year EFS outcomes (Fig. 3A) and 5 year OS outcomes (Supplementary Fig. 2A). Higher combined risk score indicated by the nomogram is associated with worse 5 year EFS outcomes, with high 21-gene signature risk score, age > 18 months at diagnosis, MYCN amplification, and INSS stage III/IV increasing a patient’s score in both EFS and OS analysis. The combined risk score demonstrated significant ability to stratify EFS (HR 6.93 [4.89–9.83], *p* < 0.0001) (Fig. 3B) and OS (HR 54.29 [20.10–146.60], *p* < 0.0001) in the pooled validation cohort (Supplementary Fig. 2B). 5 year ROC curves also demonstrated an improved ability to predict survival outcomes compared to the signature or any clinicopathological variable in isolation (EFS AUC = 0.839; OS AUC = 0.908) (Fig. 3C and Supplementary Fig. 2C). These results suggest that our 21-gene risk signature can be effectively incorporated with commonly used clinicopathological tools to improve risk stratification and survival outcome prediction in the clinic.

## Discussion

Despite dramatic advances in treatment strategies for neuroblastoma in the past decades, risk stratification continues to present a barrier to clinical care for neuroblastoma patients^28^. As a highly heterogenous tumor, stratifying those patients with aggressive disease from those with relatively indolent disease presents significant value to inform therapeutic intensification and deintensification strategies^21^. While risk stratification tools have been effective in improving patient outcomes, there continues to be patient subpopulations who may benefit from more accurate risk stratification to maximize treatment benefit and reduce morbidity associated with high treatment burden^29^. This study utilized an unbiased machine learning approach, LASSO regularized CoxPH regression, to generate and robustly validate a novel 21-gene transcriptomic signature that is able to accurately predict 5 year EFS and OS outcomes in neuroblastoma patients.

The 21-gene signature demonstrated a substantial ability to stratify EFS and OS across all cohorts studied and was capable of effectively predicting 5 year survival outcomes for patients, with AUC values in excess of 0.8 for all analyses. These findings suggest that the obtained signature has strong prognostic value and supports the ability of the 21-gene signature to provide clinically significant prognostic information. Further, the signature was capable of stratifying within several relevant subpopulations, including the MYCN unamplified clinical subgroup which demonstrates substantial prognostic heterogeneity^49^. The developed signature also retained its independent prognostic effect in multivariate analysis with clinicopathological variables, including age at diagnosis, MYCN amplification status, and INSS stage–all of which are used to varying degrees to stratify neuroblastoma patient risk. These results supports the signature’s utility in stratifying patients regardless of their clinicopathologic features at presentation. Given this strong performance, we generated an intuitive nomogram to allow for easy incorporation of risk as defined by the 21-gene signature with these clinicopathologic variables for both EFS and OS outcomes. When combined with these clinicopathological risk factors to generate a combined risk score using these tools, the predictive capacity of the signature further improved beyond that of the risk factors or prognostic signature alone–validating the synergistic benefit of combining variables and providing an improved risk stratification tool that incorporates the contribution of both clinical features and biological correlates. Of note, the combined risk score had an AUC of 0.839 and 0.908 for prediction of 5 year EFS and OS outcomes respectively, suggesting strong prognostic utility. Aside from its strong prognostic capabilities across numerous outcomes and the ease of incorporating it with existing clinicopathological variables, the 21-gene signature has the added advantage of being developed across numerous platforms. The validation cohorts utilized in this study quantified gene expression using the Affymetrix Human Exon ST 1.0 microarray and a custom Agilent microarray for the GSE85047 cohort and E-MTAB-179 cohort respectively. Few other prognostic signature studies for neuroblastoma have utilized cross-platform analyses^43^, and the high predictive utility of our signature across platforms suggests its association with survival is independent of the quantification technology used. To the best of our knowledge, the combined risk score developed in this study represents the highest predictive accuracy, in independent external validation cohorts, of a cross-platform signature for prediction of neuroblastoma EFS and OS outcomes.

There have been numerous studies which have looked to generate novel transcriptomic signatures for prognostication in neuroblastoma, incorporating a number of different statistical and methodological techniques^35–38^. Multiple signatures have been developed for use in particular subgroups of neuroblastoma, particularly among high-risk patients^50,51^. Additionally, a number of investigators have endeavoured to make biologically attuned signatures, selecting for genes associated with signaling or cell-cycle pathways such as MYCN^40^, or biological processes implicated in neuroblastoma such as those related to hypoxia^41^. These signatures have demonstrated strong prognostic performance in validation analyses and have generated new insight into the cellular mechanisms that may underly the aggressiveness of a subset of high-risk neuroblastoma cases^40,41^. Several different transcriptomic technologies have been utilized in these studies, including the use of RT-qPCR^36^and nanoString nCounter^44^ platforms which are particularly important given the ease of translating such assays into the clinical environment. Previous analyses have also varied significantly with regards to the methods used to generate signatures in their work, with stepwise CoxPH regression^52^, support vector machines^53^, and artificial neural networks^54^ being among the most widely utilized, with many showing reasonable performance in stratifying survival.

The approach used in the current study has multiple advantages that add meaningfully to this existing literature. First, we utilize a machine learning approach with no a priori selection of candidate transcripts allowing the identification and inclusion of the most relevant prognostic genes. Additionally, unlike neural network approaches, the LASSO regularized CoxPH regression utilized in this work allows for a simple mathematical model to be used to calculate patient risk enabling easier integration into clinical workflows. Additionally, our signature stratifies both OS and EFS both of which are survival outcomes that are routinely used in COG and INRG clinical trials^8^ and are central to patient and family counselling in the clinical environment. Finally, we demonstrate the independence of our signature and generate intuitive nomograms to allow for easy calculation of risk scores that incorporate our signature. Finally, the signature presented shows improved prognostic performance in validation analyses compared to many of the existing signatures described in the literature, providing incremental utility as a molecular biomarker for neuroblastoma.

Though the precise roles of many genes included in our prognostic signature remain unclear, multiple genes have previously been associated with neuroblastoma pathophysiology and prognosis. Increased expression of ECEL1 has been associated with favorable prognosis and a more benign phenotype in multiple in-vitro studies^55^ and has been included in previous prognostic signatures for neuroblastoma^35,44^. HOXC9 expression has been associated with spontaneous regression of neuroblastoma and is a positive prognostic marker for survival^56,57^. Similarly, decreased expression of GABARAP and GABAergic gene family members have been associated with poor outcome in a subset of neuroblastoma patients^58^. Additionally, DYRK3 expression has been associated with a potential role in differentiation and hypoxic control of neuroblastoma cell lines within in vitro studies^59^. Though GNA14 expression has not been strongly associated with neuroblastoma survival, somatic mutations in this gene have been associated with congenital and sporadic vascular tumors^60^. Importantly, the contribution of these genes to risk as predicted by our signature has the same directionality as the association with survival described in the literature, indicating that our findings are in line with previous work in this area. Given the association of the 21-genes identified in this study with survival they may represent a subset of functionally relevant contributors to neuroblastoma aggressiveness or host disease susceptibility. As such, further investigation of these genes, including those with positive contribution to risk score (putative oncogenes) or those with negative contribution to risk score (putative tumour suppressor genes) may help further characterize mechanisms underlying the heterogeneity of neuroblastoma outcomes.

This study used three independent datasets for which follow-up, clinicopathologic, and expression data was available to develop a strong predictor of EFS and OS outcomes in neuroblastoma patients. Our approach was not biased by any a priori biological insight, and therefore the candidate gene set was not restricted for this reason, allowing for the most significant prognostic genes to be selected. However, the pool of candidate genes was restricted in this analysis to those quantified by all three cohorts selected. While a large selection of genes was included in this analysis, the exclusion of genes that were not present across all cohorts may have removed potentially significant candidates from signature development. Additionally, multivariate analysis was also restricted to variables present across all three cohorts. Therefore, potentially useful factors such as patient sex, INRGSS stage, and COG risk classification could not be effectively incorporated into the nomogram for combined analysis. Despite this, our signature still has substantial prognostic utility and future work should attempt to incorporate the presented signature with other clinicopathological and transcriptomic prognostic markers to further improve its performance.

An increasing number of transcriptomic signatures are being translated into the clinical environment, further demonstrating the utility and translatability of molecular biomarkers. In particular, multiple gene signatures for guidance of treatment selection and prediction of relapse have been validated and are in clinical use in the field of breast cancer^61,62^, as are tools for more accurate identification of cancerous nodules in the area of thyroid cancer^63^. Prior to the implementation of the identified 21-gene risk signature into clinical settings, a reliable quantification assay must be developed and validated. This could include either real-time PCR or targeted next-generation sequencing-based methods. Given that the signature has been validated across multiple expression platforms, it is likely that any of these technologies may be utilized successfully. Following these studies, prospectively testing this signature in large multi-centre clinical trials will be paramount to incorporate it as a prognostic tool into neuroblastoma risk stratification and management. In summary, our combined prognostic signature may help identify a subset of patients with poor prognosis that are candidates for treatment intensification and close monitoring.

## Supplementary Information

Supplementary Information.

## Data Availability

Gene expression and clinical data included in this study was obtained from the following publicly available datasets: Therapeutically Applicable Research to Generate Effective Treatments (TARGET) initiative (sub-study ID phs000467), Array Express (Accession E-MTAB-179), and the Gene expression Omnibus (Accession GSE86047). All other data relevant to the study will be made available upon reasonable request to corresponding authors.
