# Supplementary material for: Development and validation of a 21-gene prognostic signature in neuroblastoma
Source: Sci Rep. 2023 Aug 2;13:12526. doi: 10.1038/s41598-023-37714-9 (PMC10397261; doi:10.1038/s41598-023-37714-9)
Supplement: Supplementary file 1 — Supplementary Information. [file 41598_2023_37714_MOESM1_ESM.docx]

**Supplementary Table 1.** **Component genes included within the 21-gene prognostic signature.** Genes are identified by the HUGO gene symbol and name, as well as chromosomal location. Signature association is an indication of if increased expression of the gene is associated with good prognosis (negative) or poor prognosis (positive). Regression coefficients demonstrate the relative contribution of each gene to the overall 21-gene risk score.

| **Gene Symbol** | **Gene Name** | **Chromosome** | **Location (bp)** | **Signature Association** | **Regression Coefficient** |
| --- | --- | --- | --- | --- | --- |
| CILK1 (ICK) | ciliogenesis associated kinase 1 | chr6 | 53061824-53001299 | Negative | -0.4822725 |
| ECEL1 | endothelin converting enzyme like 1 | chr2 | 232487834-232479827 | Negative | -0.2246051 |
| ARRB1 | arrestin beta 1 | chr11 | 75351662-75260122 | Negative | -0.1599832 |
| HOXC9 | homeobox C9 | chr12 | 54000161-54003337 | Negative | -0.1409085 |
| GPR85 | G protein-coupled receptor 85 | chr7 | 113087724-113081854 | Negative | -0.1281612 |
| APAF1 | apoptotic peptidase activating factor 1 | chr12 | 98645141-98735433 | Negative | -0.0992775 |
| GPR68 | G protein-coupled receptor 68 | chr14 | 91264581-91232532 | Negative | -0.0434883 |
| GABARAP | GABA type A receptor-associated protein | chr17 | 7242449-7240008 | Negative | -0.0372841 |
| ENTPD3 | ectonucleoside triphosphate diphosphohydrolase 3 | chr3 | 40387182-40428744 | Negative | -0.0181622 |
| ALDH3A2 | aldehyde dehydrogenase 3 family member A2 | chr17 | 19648136-19677596 | Negative | -0.0139724 |
| CBLN1 | cerebellin 1 precursor | chr16 | 49281838-49277917 | Positive | 0.0255775 |
| MX2 | MX dynamin like GTPase 2 | chr21 | 41361944-41409393 | Positive | 0.0741968 |
| ADM | adrenomedullin | chr11 | 10305073-10307397 | Positive | 0.0800572 |
| DYRK3 | dual tyrosine regulated kinase 3 | chr1 | 206635536-206655158 | Positive | 0.1005547 |
| GNA14 | G protein subunit alpha 14 | chr9 | 77648322-77423079 | Positive | 0.1683114 |
| CPT1B | carnitine palmitoyltransferase 1B | chr22 | 50578612-50568861 | Positive | 0.1852795 |
| CRB1 | crumbs cell polarity complex component 1 | chr1 | 197201504-197478455 | Positive | 0.2251831 |
| FTSJ3 | FtsJ RNA 2'-O-methyltransferase 3 | chr17 | 63827663-63819433 | Positive | 0.2327809 |
| HOMER2 | homer scaffold protein 2 | chr15 | 82986176-82836946 | Positive | 0.2863938 |
| MTNR1A | melatonin receptor 1A | chr4 | 186555567-186532769 | Positive | 0.3934321 |
| IRAK1 | interleukin 1 receptor associated kinase 1 | chrX | 154019984-154010507 | Positive | 0.7447583 |

**Supplementary Table 2.** **Univariate cox proportional hazards analysis of overall survival for high vs. low-risk groups as defined by the 21-gene prognostic signature in clinically relevant subgroups.** Subgroups were stratified by age at diagnosis (<18 months and >18 months groups), INSS stage (I, II and III, IV groups) and MYCN amplification status (amplified and unamplified groups). Dichotomization of patients into high and low-risk groups was performed using the median prognostic signature score.

|  |  | **HR (95% CI)** | **p-value** |
| --- | --- | --- | --- |
| **GSE85047** *(N=240, events = 72)* | Age at Diagnosis  (>18 Months)  *n= 126, events= 60* | 1.873  (1.111 – 3.159) | **0.0185** |
|  | Age at Diagnosis  (<18 Months)  *n= 114, events= 12* | 5.786  (1.267 - 26.420) | **0.0235** |
|  | INSS Stage  (III, IV)  *n= 163, events= 70* | 2.429  (1.479 - 3.990) | **<0.0001** |
|  | INSS Stage  (I, II)  *n= 77, events= 2* | 1.043  (0.065 – 16.69) | 0.9760 |
|  | MYCN  Amplified  *n= 53, events= 35* | 1.014  (0.521 – 1.974) | 0.9670 |
|  | MYCN  Unamplified  *n= 187, events= 37* | 6.106  (2.671 – 13.960) | **<0.0001** |
| **E-MTAB-179**  *(N=416, events = 87)* | Age at Diagnosis  (>18 Months)  *n= 168, events= 74* | 2.818  (1.733 – 4.582) | **<0.0001** |
|  | Age at Diagnosis  (<18 Months)  *n= 248, events= 13* | No Events in Low-Risk Group | No Events in Low-Risk Group |
|  | INSS Stage  (III, IV)  *n= 217, events= 115* | 5.353  (3.149 – 9.100) | **<0.0001** |
|  | INSS Stage  (I, II)  *n= 199, events= 7* | No Events in Low-Risk Group | No Events in Low-Risk Group |
|  | MYCN  Amplified  *n= 64, events= 41* | 1.215  (0.656 - 2.249) | 0.5350 |
|  | MYCN  Unamplified  *n= 352, events= 46* | 53.220  (7.334 – 386.200) | **<0.0001** |

**Supplementary Table 3.** **Univariate cox proportional hazards analysis of Event Free Survival for high vs. low-risk groups as defined by the 21-gene prognostic signature in clinically relevant subgroups of the pooled validation cohort.** Subgroups were stratified by age at diagnosis (<18 months and >18 months groups), INSS stage (I, II and III, IV groups) and MYCN amplification status (amplified and unamplified groups) and iteratively combined into combinations based on the presence of multiple prognostic variables. Dichotomization of patients into high and low-risk groups was performed using the median prognostic signature score.

|  | | **HR (95% CI)** | **p-value** |
| --- | --- | --- | --- |
| **Pooled Validation Cohort**  *(N=656, events=234)* | Age at Diagnosis >18 Months  and  MYCN Amplified  *n= 86, events = 61* | 0.8804  (0.2755 - 2.814) | 0.8300 |
|  | Age at Diagnosis >18 Months  and  INSS Stage III, IV  *n= 241, events = 155* | 1.952  (1.193 - 3.195) | **0.0078** |
|  | MYCN Amplified  and  INSS Stage III, IV  *n= 109, events = 78* | 1.066  (0.4304 - 2.639) | 0.8900 |
|  | MYCN Amplified,  INSS Stage III, IV  and  Age at Diagnosis <18 Months  *n=* *81 , events = 57* | 1.292  (0.3146 - 5.302) | 0.7220 |
|  | Age at Diagnosis <18 Months  and  MYCN unamplified  *n= 331, events = 47* | 2.549  (1.416 - 4.592) | **0.0018** |
|  | Age at Diagnosis <18 Months  and  INSS Stage I, II  *n= 223, events = 24* | 3.012  (1.318 - 6.885) | **0.0090** |
|  | MYC unamplified  and  INSS Stage I, II  *n= 268, events = 29* | 3.14  (1.514 - 6.509) | **0.0021** |
|  | MYC unamplified  INSS Stage I, II  and  Age at Diagnosis <18 Months  *n= 220, events = 23* | 2.772  (1.175 - 6.539) | **0.0199** |

**Supplementary Table 4.** **Univariate cox proportional hazards analysis of Overall Survival for high vs. low-risk groups as defined by the 21-gene prognostic signature in clinically relevant subgroups of the pooled validation cohort.** Subgroups were stratified by age at diagnosis (<18 months and >18 months groups), INSS stage (I, II and III, IV groups) and MYCN amplification status (amplified and unamplified groups) and iteratively combined into combinations based on the presence of multiple prognostic variables. Dichotomization of patients into high and low-risk groups was performed using the median prognostic signature score.

|  | | **HR (95% CI)** | **p-value** |
| --- | --- | --- | --- |
| **Pooled Validation Cohort**  *(N=656, events=159)* | Age at Diagnosis >18 Months  and  MYCN Amplified  *n= 86, events = 57* | 1.94  (0.4727 - 7.966) | 0.3580 |
|  | Age at Diagnosis >18 Months  and  INSS Stage III, IV  *n= 241, events = 127* | 2.278  (1.282 - 4.047) | **0.0050** |
|  | MYCN Amplified  and  INSS Stage III, IV  *n= 109, events = 73* | 1.064  (0.4288 - 2.641) | 0.8930 |
|  | MYCN Amplified,  INSS Stage III, IV  and  Age at Diagnosis <18 Months  *n=* *81 , events = 54* | 1.46  (0.3548 - 6.006) | 0.6000 |
|  | Age at Diagnosis <18 Months  and  MYCN unamplified  *n= 331, events = 6* | 3.851  (0.7772 - 19.08) | 0.0987 |
|  | Age at Diagnosis <18 Months  and  INSS Stage I, II  *n= 223, events = 2* | 5.007  (0.313 - 80.09) | 0.2550 |
|  | MYC unamplified  and  INSS Stage I, II  *n= 268, events = 6* | 7.66  (2.058 - 151.5) | **0.0088** |
|  | MYC unamplified  INSS Stage I, II  and  Age at Diagnosis <18 Months  *n= 220, events = 2* | 5.247  (0.3281 - 83.93) | 0.240 |

**Supplementary Table 5.** **Univariate and multivariate cox proportional hazards analysis of clinicopathologic variables and 21-gene risk score for overall survival.** Patients were dichotomized by age at diagnosis (<18 months and >18 months), INSS stage (I, II and III, IV) and MYCN amplification status (amplified and unamplified groups). Dichotomization of patients into high and low-risk groups was performed using the median prognostic signature score.

|  |  | **Univariate** | | **Multivariate** | |
| --- | --- | --- | --- | --- | --- |
|  |  | **HR**  **(95% CI)** | **p-value** | **HR**  **(95% CI)** | **p-value** |
| **GSE85047** *(N=240, events = 72)* | Age at Diagnosis  *(>18 Months vs <18 Months)* | 6.547  (3.499 - 12.250) | **<0.0001** | 2.407  (1.227 – 4.720) | **0.0106** |
|  | INSS Stage  *(III, IV vs I, II)* | 23.73  (5.809 - 96.900) | **<0.0001** | 9.084  (2.090 - 39.477) | **0.0033** |
|  | MYCN Status  *(Amplified vs Unamplified)* | 4.919  (3.070 - 7.880) | **<0.0001** | 2.191  (1.333 – 3.602) | **0.0020** |
|  | 21-gene risk score  *(High vs Low)* | 4.941  (2.823 - 8.647) | **<0.0001** | 1.697  (0.907 - 3.172) | 0.0978 |
| **E-MTAB-179**  *(N=416, events = 87)* | Age at Diagnosis  *(>18 Months vs <18 Months)* | 10.460  (5.791 - 18.880) | **<0.0001** | 2.291  (1.216 – 4.315) | **0.0102** |
|  | INSS Stage  *(III, IV vs I, II)* | 11.970  (5.530 - 25.930) | **<0.0001** | 3.552  (1.589 - 7942) | **0.0020** |
|  | MYCN Status  *(Amplified vs Unamplified)* | 9.174  (5.974 - 14.090) | **<0.0001** | 2.894  (1.863 – 4.495) | **<0.0001** |
|  | 21-gene risk score  *(High vs Low)* | 37.240  (11.760 - 117.900) | **<0.0001** | 10.990  (3.243 – 37.241) | **<0.0001** |

**Supplementary Figure 1.** Overall survival stratified by 21-gene prognostic signature risk score in discovery and validation cohorts. Kaplan Meier survival curves with cox proportional hazards analysis showing risk of overall survival in low risk (black curve) and high risk (grey curve) groups generated using a median cut-off for the 21-gene risk score and ROC curve analysis demonstrating the predictive capacity of the risk score for 5-year overall survival in the discovery cohort (Panel A and B), GSE85047 validation cohort (Panel C and D), and E-MTAB-179 validation cohort (Panel E and F).

**
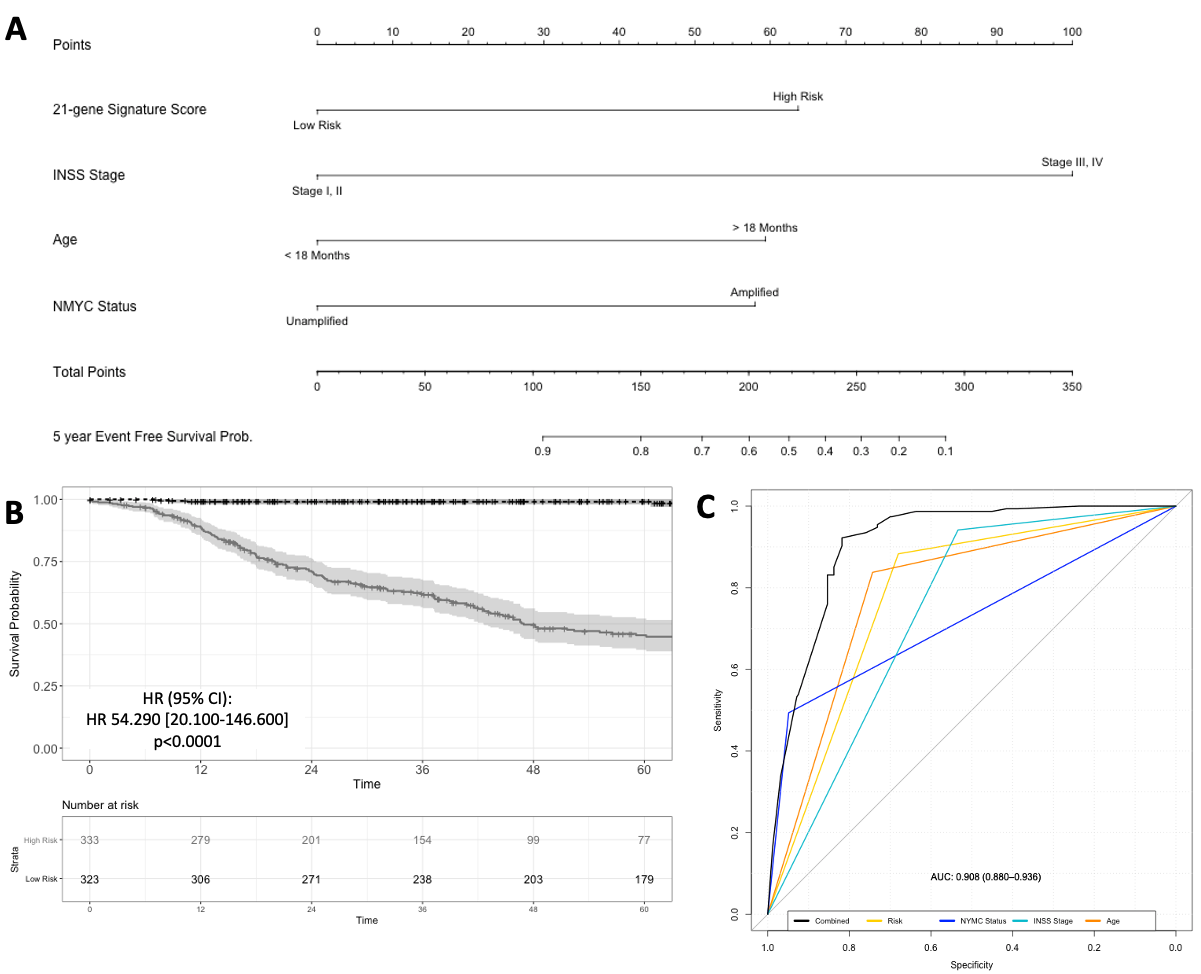
**

**Supplementary Figure 2.** Nomogram integrating 21-gene risk score and other clinicopathological variables for prediction of 5-year overall survival in a pooled validation cohort. Nomogram incorporating clinicopathological characteristics and 21-gene risk score to generate a combined risk score (Panel A). Kaplan Meier survival curves with cox proportional hazards analysis showing risk of overall survival in low risk (black curve) and high risk (grey curve) groups generated using a median cut-off for the combined prognostic risk score in the pooled validation cohort (Panel B). ROC curve analysis demonstrating the predictive capacity of the combined risk score compared to the 21-gene risk score and other clinicopathological variables in isolation for 5-year overall survival in the pooled validation cohort (Panel C).
